# The Arts Therapies in Palliative and End-of-Life Care: Insights from a Cross-Cultural Knowledge Exchange Forum

**DOI:** 10.3390/bs15050602

**Published:** 2025-05-01

**Authors:** John F. Mondanaro, Bruce Armstrong, Sally McRae, Edith Meyerson, Todd O’Connor, Giorgos Tsiris

**Affiliations:** 1 The Brookdale Department of Geriatrics and Palliative Medicine, Icahn School of Medicine at Mount Sinai, New York, NY 10029, USA; edith.meyerson@mssm.edu; 2 St Columba’s Hospice Care, Edinburgh EH5 3RW, UK; barmstrong@stcolumbashospice.org.uk (B.A.); smcrae@stcolumbashospice.org.uk (S.M.); gtsiris@stcolumbashospice.org.uk (G.T.); 3 Mount Sinai Kravis Children’s Hospital, New York, NY 10029, USA; 4 Division of Occupational Therapy and Arts Therapies, Queen Margaret University Edinburgh, Edinburgh EH21 6UU, UK

**Keywords:** palliative and end-of-life care (PEoLC), arts therapies, interdisciplinary, knowledge exchange

## Abstract

In October 2023, a Knowledge Exchange Forum was established, bringing together arts therapies staff and students from three different palliative and end-of-life care (PEoLC) settings: St Columba’s Hospice Care in Edinburgh, the Brookdale Department of Geriatrics and Palliative Medicine at Mount Sinai Hospital and the Mount Sinai Kravis Children’s Hospital in New York. Adopting a practice-led approach, the Forum offers a space to unpack questions and challenges that arts therapists face in PEoLC. In this dialogical paper, we outline the development of the Forum and share emerging insights from our cross-cultural discussions. When working in PEoLC settings, arts therapists are commonly required to work across a continuum of care. This continuum extends from individual sessions with patients, families and bereaved carers, to groups and community-oriented initiatives. It often requires a capacity to work flexibly and fluidly with regard to, for example, therapeutic boundaries, consistency of location, and time. Discussion of emerging insights leads to a consideration of their implications for education and practice, and for future directions in professional networking and knowledge exchange.

## 1. Introduction

Palliative and end-of-life care (PEoLC) has grown rapidly in the UK, USA and other Western countries since the birth of the modern hospice movement in the late 1960s (Clark et al., 2020). Dame Cicely Saunders, the founder of the modern hospice movement, advocated the view that “You matter because you are you, and you matter to the end of your life. We will do all we can not only to help you die peacefully, but also to live until you die”. Her vision is still at the heart of contemporary palliative care, and it sets the foundations for a person-centered approach to care. In part, this approach entails a shift of focus from “what’s the matter” to “what matters to you”, so that the person is viewed in their totality. Saunders’ concept of ‘total pain’ remains fundamental to palliative care, highlighting not only the physical, but also the social, emotional and spiritual pain that the person experiences when living with an incurable illness (Wood, 2022). This person-centered and holistic approach underpins PEoLC, and it has fostered a fertile environment for cross-professional and interdisciplinary collaboration over the past decades. This dialogical paper aims to share emerging insights from a Cross-Cultural Knowledge Exchange Forum for arts therapies in PEoLC in Edinburgh (UK) and New York (USA). We discuss the need for knowledge transfer and exchange in palliative care, and outline the development of the Forum while positioning ourselves and introducing our respective settings. Reflecting on insights emerging from our dialogues to date, we consider potential implications for PEoLC practice and education in arts therapies. To set the wider context, however, we first reflect on the arts therapies in PEoLC and the importance of relational work.

### 1.1. Arts Therapies in Palliative and End of Life Care (PEoLC)

Arts and creativity have been an integral part of the modern palliative care movement throughout its history. For example, there are early accounts of musicians playing in palliative care inpatient units, or in day programs offering patients opportunities for creative engagement. Yet the establishment of arts therapies as distinct professional practices facilitated by qualified arts therapists has a shorter history, and their establishment as an integral part of palliative care service provision has faced certain challenges. Some of these challenges pertain to the lack of palliative care-specific education for arts therapists. Hartley (2008) has questioned, for example, to what extent generic arts therapies education in the UK promotes a specific way of thinking (e.g., around therapeutic boundaries) that may be at odds with the realities and demands of PEoLC work.

The diversity and uniqueness of arts therapy work in PEoLC settings is shaped by the very nature of the work, which has death, dying, and loss at its heart. The unique features of arts therapy practice in this context are related to factors including people’s fluctuating health conditions, time limits due to the typically short-term nature of the work, variations in session locality and format, and the diversity of referral reasons.

The integration of arts therapies into PEoLC and their relational focus are well established in the literature (Clements-Cortes & Yip, 2021; Schmid et al., 2018; Warth et al., 2015, 2019). Arts-based therapies are variously defined in the literature, and variations in terminology and designation move beyond matters of semantics (Dunphy et al., 2014). *Creative Arts Therapies* as a term may foreground the unique capacity of creativity as an innate human experience integral to health and healing. *Expressive Arts Therapies* assert the essential role of expressivity to the human experience in states of wellness and illness, and commonly promote the integration of different arts modalities and cross-media work. Embracing both creativity and expressivity, *Arts Therapies* is perhaps the most commonly used umbrella term that includes different, yet interrelated, professional fields: music therapy, dramatherapy, art therapy, and dance movement therapy. This diversity of terminology contributes to the colorful landscape of the arts in palliative care (Tsiris & Lee, in press), highlighting the diversity of underpinning theoretical approaches, sociocultural influences, and professional currents that exists in this field (Karkou et al., 2017). In this paper, for consistency, we use the term ‘arts therapies’. We use the term ‘patient’ to describe a person who lives with an incurable illness and accesses PEoLC.

A large part of the arts therapies literature to date focuses on individual work, often informed by psychodynamic principles. Yet, such literature trends do not always seem correspondent with practice developments in the field. For example, the focus on individual work through a psychodynamic lens is only one (albeit important) aspect of arts therapists’ work. Many arts therapists work with closed and open groups, and some facilitate community-oriented projects with potential public and performance elements, within a public health and health-promoting PEoLC framework. 

The diversity of such practices resonates with the ‘continuum of care’ described by Tsiris et al. (2022b). The legitimization, prioritization and implementation of this diversity depends on the dynamics of each context of practice, shaped by professional agendas and dominant narratives in the field, by organizational priorities and funding, and by national strategies and policies. In all cases, however, arts therapies practices are fundamentally relational. Equally, this emphasis on relational work is a bedrock of PEoLC more broadly.

### 1.2. Relational Work

The underpinning philosophy of PEoLC disrupts a long-held medical narrative focused on the domain of the body by embracing a holistic approach to care. Providing whole-person—body, mind, and spirit—care that is relational entails challenges for practitioners working in the context of institutional and systemic obstacles such as time constraints, administrative limitations, and funding issues. Despite the positive effect of PEoLC on quality of life and symptom burden (Kavalieratos et al., 2016; Radbruch et al., 2020; Rosenberg et al., 2021), the impact of such issues can be significant (Busolo & Woodgate, 2016; Crawford et al., 2013; Evans et al., 2019; Harasym et al., 2020). Exploring this concern, Glasdam et al.’s (2020) study showed that administrative structures and role delineation in palliative care settings prevented nurses from realizing their own humanistic values in caring for patients. Such obstacles to relational care in nursing may also affect other healthcare providers, including arts therapists. Attempts to mitigate the impact of such obstacles by realigning focus on the patient include Österlind and Henoch’s (2021) 6S-Model, which centralizes the patient’s self-image as the entry point to assessment and treatment. They emphasize symptom support, self-determination, social relationships, and the synthesis of experiences, which function collectively in varying degrees to serve a person-centered palliative care ethos. This ethos holds equal resonance in the approaches of arts therapists in PEoLC. In a similar vein, McLaughlan et al. (2022) gathered responses from 89 Australian-based palliative care professionals who found that the actual physical environment, including space beyond patient rooms such as gardens, helped manifest the quality of care hoped for by those working with patients. The aesthetic qualities of an environment that invites emotional connection and supports morale contribute to the idea of relational work and aesthetic experience—the milieu of arts therapies—as being a core aspect of both patient care and staff satisfaction in PEoLC.

The convergence of mental healthcare with PEoLC raises questions regarding how psychotherapeutic practice pertains to the emotional support of patients who are dealing with serious physical illness. Apropos, Rosenberg et al. (2021) make a strong case for the therapeutic relationship in the delivery of care, and delve into the psychotherapeutic dynamics of transference and countertransference as important educational points for palliative clinicians historically untrained in or unfamiliar with psychotherapeutic techniques. Their writing highlights casework demonstrating the effectiveness of relational work in PEoLC. Looking beyond the experience of palliative care clinicians, Park et al. (2022) look at that of mental health professionals working in palliative care, revealing gaps in training regarding areas such as intersectionality, death and dying, unconscious bias, and relational work itself. 

PEoLC requires a relational approach but this can be difficult to achieve in some settings where, to varying degrees, there may be reportable gaps in the education of professionals working in palliative care as well as of mental health clinicians consulting on palliative cases. The result of this can be that the task of relational work is shouldered primarily by interdisciplinary team members such as arts therapists, chaplains, and social workers, who bring a diverse skill set that includes mental health training. Relevant to this, Orkibi et al. (2023) conducted a three-year study on the impact of training specific to PEoLC, finding a need for such resources specifically designed for arts therapists. This need has been long recognized for arts therapists working in PEoLC settings. In addition to established models and resources, such as institute training through Dr. Russell Hilliard’s Center for Music Therapy in End of Life Care, supervision has also been cited as essential (Potash et al., 2015), as is continuing education and immersion in such areas as diversity, inclusion and equity (DEI) awareness and cultural humility (Singh et al., 2023). 

Arts therapies practitioners may be required to navigate the unique features of PEoLC practice, while also experiencing some degree of professional isolation, often exacerbated by part time employment or by being the sole arts therapies practitioners within an entire organization (Drury & Tsiris, 2022). This highlights the need for professional networking and knowledge exchange opportunities in the field. In recent years, specific forums and networks have emerged, such as an Arts in Palliative Care Community of Practice and a subsequent Extension of Community Healthcare Outcomes (ECHO) Network (an online network to support Scotland-based arts therapists and arts practitioners working in palliative care), and the development of professional events dedicated to the arts in PEoLC, such as the Facing Death Creatively conference (St Christopher’s Hospice, London) and the International Annual Symposium for the Arts in Palliative Care (St Columba’s Hospice Care, Edinburgh). Learning more about such initiatives can inform future practice, education and theory development in the field. To this end, the present paper focuses on the establishment of a Cross-Cultural Knowledge Exchange Forum for arts therapies in PEoLC.

## 2. The Knowledge Exchange Forum

Our Knowledge Exchange Forum was formed in October 2023 by G.T. and J.M. Earlier collaborative work between G.T., J.M. and other international colleagues regarding relational and community perspectives in palliative care (Clements-Cortes et al., 2022, 2023) had highlighted the need for increased cross-cultural and organizational dialogue and offered the impetus for this initiative.

Bringing together arts therapies staff and students from the three respective settings, the Forum adopts a practice-led approach. It focuses on participants’ lived experience of arts therapies practice and education, and seeks to offer a space for peer reflection and learning with a particular emphasis on students’ experience. The cross-cultural nature of the Forum is crucially important, as it allows reflection on how the sociocultural context of each setting influences practice and service developments. The Forum also has a pedagogical aspect, in that its membership includes palliative care professionals with a practice education and supervisory responsibility for students, as well as professionals with a teaching role. Students are invited to share casework or aspects of their experience as learners within Forum sessions, and although these contributions are not formally assessed, they occur within practice education and supervisory relationships that exist between Forum members. It should also be noted that none of us as an author is a student (although S.M. was formerly a student member of the Forum). An important aim of the Forum meetings is to afford a space where students across the three settings can share their experiences and insights with each other. The resulting discussions are typically free-ranging and dialogical, and are supported by contributions from the staff members of the group. The Forum meetings take place online through video conferencing once a month and are structured according to the academic year. The first cycle of meetings took place between October 2023 and March 2024. Following informal evaluation and debrief, we developed the second cycle of meetings (October 2024–March 2025).

The meetings are co-facilitated by the authors for creative dialoguing on topics that define PEoLC. Although E.M. does not attend the meetings, she provides supervisory support for J.M., which is filtered back to the group in the form of insights for consideration. Inviting all voices to ask questions and share their own experience through case study discussions is essential for learning and contributes richly to a learning community (R. E. Meyerson et al., 2012). Indeed, this Forum initiative has, since its inception, inspired ongoing dialogues amongst ourselves *en masse* and in various smaller configurations.

Discussions are held within a forum in which safety is ensured, as the work of arts therapists in caring for patients receiving PEoLC is shared. In the process, themes worthy of contemplation are revealed, as discussed in Emerging Insights, below. Before turning to these we position ourselves and our respective settings.

### 2.1. Positioning Ourselves and the Three Settings

The arts therapies are a heterogeneous field underpinned by different theoretical frameworks and ways of practicing. Such differences have given rise to diverse approaches within each arts therapies profession, including but not limited to psychodynamic, community and developmental approaches.. Although such differences are reflected across the membership of the Forum, we all operate within a predominantly Western paradigm of healthcare, with some shared understandings of health, illness, and palliative care. Both in the USA and UK, arts therapies are established as regulated professions underpinned by certain standards of proficiency and professional codes of ethics. In both countries, the provision of arts therapies requires a certified professional who has completed an approved education program. In the UK, arts therapies education is offered only on a master’s level and all students are required to engage in personal psychotherapy for a minimum number of hours during their training. Both in the USA and in the UK, arts therapies students are required to undertake practice-based learning in the form of student placements or internships under supervision. Such supervision is typically offered by an experienced therapist who serves as practice educator or supervisor.

The differences between our respective contexts offer a rich learning platform. Such differences relate to the sociocultural, political and health economy landscapes of the UK and USA, as well as to our varied palliative care provision contexts (i.e., hospice, palliative care hospital unit, and acute end-of-life care children’s hospital provision), as outlined below.

#### 2.1.1. The Arts Team at St Columba’s Hospice Care, Edinburgh

Established in 1977, St Columba’s Hospice Care is the first modern hospice in Scotland. Its catchment area, extending across North Edinburgh and East Lothian, includes approximately 400,000 people. The hospice’s mission is to support and empower people living with and dying from life-limiting illnesses, and those close to them. St Columba’s is an independent charitable organization and all its services are offered free-of-charge. The hospice collaborates closely with other service providers within and beyond the National Health Service, but the funding of its services depends largely on donations.

In addition to offering specialist multi-professional PEoLC and bereavement support, the hospice supports people to live as independently as possible. With a balanced focus between inpatient and outpatient services and an emphasis on community development, the hospice also adopts a health promoting stance aiming to create communities which are more comfortable with dying, death and bereavement (St Columba’s Hospice Care, 2024).

The arts team of the hospice is an integral part of its service provision, navigating a spectrum of specialist clinical practices and community-oriented ones. The team consists of arts therapists and community artists, offering a range of sessions and projects to support patients, families and the local community by helping them to explore and make sense of their life experiences, as well as to express themselves and connect with each other creatively using different arts media. Arts therapies practices can vary in configuration, depending on people’s needs, and may include individual private sessions (at the bedside or at our dedicated Art Studio or Music Studio), closed groups and open group work, as well as community-oriented work.

As a University Hospice, St Columba’s hosts placements of music, art and dramatherapy students from MSc programs at Queen Margaret University (QMU), in Edinburgh. Being the only arts therapies education provider in Scotland, QMU programs promote a person-centered approach with a strong emphasis on psychodynamic thinking. In recent years, engagement with issues regarding equality, diversity, inclusion and belonging (EDIB) have shaped the arts therapies programs’ curricula and ways of working (Tsiris et al., 2022a), while complying with the professional standards set by the Health and Care Professions Council (HCPC); the regulatory body for arts therapists in the UK.

#### 2.1.2. The Expressive Arts Therapy Program Within the Brookdale Department of Geriatrics and Palliative Medicine, Icahn School of Medicine, Mount Sinai Hospital, New York

The Expressive Arts Therapy Program was founded in 2022 within the Brookdale Department of Geriatrics and Palliative Medicine, which is housed in the Icahn School of Medicine of Mount Sinai Hospital in New York. The department offers extensive programming across inpatient and outpatient milieus. The expressive Arts Therapy program is focused on inpatient care, with attention given to three consultation teams across the medical center’s medical units and intensive care units, a primary palliative care unit, and palliative programs embedded in cardiology, neurology, and the medical intensive care unit. 

While the Brookdale Department has long valued interdisciplinary care through its integration of art therapy, chaplaincy, social work, massage therapy, and yoga therapy, this particular incarnation was formed with the philosophy that music, art, dance-movement, and drama therapies would reside alongside child life under the relational umbrella of Expressive Arts Therapy (Mondanaro, 2024, 2025). Representation of each art modality enhances the care of patients through its affordance of multiple therapeutic entry points. Child life, typically located in pediatric hospitals, is focused here on children and teens reconciling the loss of a significant adult receiving PEoLC (Mondanaro, 2024, 2025; Mondanaro & Sturgeon, 2022). The founding director, dually certified in music therapy and child life, addresses these needs through a family systems lens, utilizing developmentally targeted psychoeducational narrative therapy to support inclusion and authentic communication (Mondanaro, 2025).

Expressive arts therapy goals include fostering and enhancing expressivity, identity affirmation, aesthetic framing of medical circumstances, supporting anticipatory grief, and coping support specific to such existential themes as the myriad forms of loss, separation, change, transition, and betrayal. Embodied work in the form of tension release, pain coping, and meditative breathwork are rendered across modalities as well.

Internship opportunities for graduate level students studying any one of the four arts therapies named above are offered and supervised by the founding director. Students work as integrated members of the palliative team’s core interdisciplinary team, accruing the necessary hours to sit for their respective credentialing boards which, for some, is the first step toward licensure as a creative arts therapist in the state of New York. Augmenting the training and supervision, arts therapies students are invited to deepen their immersion in the philosophy of palliative care through their completion of online modules covering topics ranging from values focused communication to understanding diagnoses and symptom management. These modules are made available through the Center to Advance Palliative Care. 

#### 2.1.3. Creative Arts Therapies at the Mount Sinai Kravis Children’s Hospital, New York

The Mount Sinai Kravis Children’s Hospital is a long-standing pediatric hospital within the Mount Sinai Health System, which is situated in New York City and extends to its surrounding areas. In the 1960s, dimensions of therapeutic play, and practices that were becoming core to the then-emerging child life profession, were integrated into pediatric care at Kravis. Creative arts therapies additionally followed, beginning with an art therapy position in the mid-1970s, and are now an essential component of programming provided by the Child Life and Creative Arts Therapy Department. Today, a robust team of child life specialists; art, music, and drama therapists; an animal-assisted therapy program; and video arts specialists who oversee a broadcast studio (KidZone TV) within the hospital serve as a core to our broader programming.

Within our medical/surgical setting, creative arts therapists address a broad range of needs presented by patients and families, beginning with bolstering their capacity for adjustment and coping with aspects of hospitalization, medical treatment, and disease process, and extending to ongoing supportive accompaniment as they experience the fraying of normal routines, changing sense of self, and their roles within familial relationships and their community, as well as the anxiety, fear, and pain that can define their daily experience. While the Kravis Children’s Hospital is not a palliative care facility, end-of-life care and bereavement support is a domain of care our team provides in collaboration with the medical team, social work, spiritual care, and other specialists within the inpatient environment. Although creative arts therapies students at this site are not afforded the same degree of experience and exposure to EoLC as other student colleagues within our knowledge exchange, themes pertaining to loss and grief are central to their work, and these are explored throughout their internship experience as well as in the Forum discussed in the present article.

## 3. Emerging Insights

During Forum discussions, a range of key thematic areas gained salience in relation to PEoLC practice. The themes discussed below pertain to the group’s observation of therapeutic boundaries and countertransference, neutrality, curiosity, disclosure, safety of the therapeutic space, authenticity, fallibility of the therapist, and power dynamics. These themes were explored relative to the experiences of staff and students while considering potential overlaps and differences between our distinct palliative care environments—hospice care, inpatient hospital-based palliative care, and pediatric medical/surgical hospital care—and our respective sociocultural settings.

Our writing below draws on and resembles the conversational nature of our Forum discussions. We cite literature to acknowledge and support certain key ideas, but our focus is on the immediacy of the thinking that emerged in our dialogues. To this end, we offer reflections and brief vignettes that have been edited and composed by drawing on our notes and discussions. We aim to convey perceptions, opinions, beliefs, and attitudes ensuant from our conversations across the Forum meetings, together with reflections made in the interims.

### 3.1. Boundaries and Disclosure

In-depth understanding and use of therapeutic boundaries is a core component of arts therapies and often distinguishes them from other arts-based practices. In our discussions, this was particularly evident in the context of St Columba’s Hospice Care, where arts therapists navigate their practice along a wide continuum of arts practices and provisions, such as community art and creative cultural work.

Therapeutic boundaries, as a feature of psychotherapy more generally, set certain practical, relational and ethical parameters and limits. Some boundaries, such as consistency of session location and time, form part of the therapeutic setting (O’Neill, 2007). Boundaries contribute to a safe framework for the therapeutic process, supporting and protecting both the client and the therapist. Holding therapeutic boundaries often translates into practical considerations such as not accepting gifts, not having out-of-session contact and ensuring confidentiality (e.g., social media policy). 

In acknowledging that an understanding of therapeutic boundaries is of paramount importance in arts therapies education, our Knowledge Exchange Forum discussions have highlighted the need for critical and flexible engagement that considers the specificities of PEoLC. When working with people in PEoLC, a flexible approach is often needed. The session space, for example, may need to change over time, from a dedicated arts therapies room to the person’s bedside, as their condition deteriorates. Similarly, a more flexible approach may be needed regarding the time and duration of the sessions, depending on the person’s changing needs. This may seem obvious to a seasoned palliative care practitioner, but students often benefit from clear support regarding the nuanced understanding and application of boundaries, especially perhaps during their early endeavors to apply general therapy constructs within a palliative care setting.

Vignette by arts therapies student

Due to a decline in wellbeing and becoming bedbound in the inpatient unit, a regular dramatherapy group member was unable to attend a session. The dramatherapy student had built good rapport with the patient over the preceding weeks. Whilst the student was attending the patient bedside, the patient asked for a drink. The student assisted them in sipping a glass of water. The act of holding the glass to the patient’s lips, felt appropriate yet highly intimate—akin to feelings of supporting a loved one.

The example above prompted curiosity and discussion in the Forum. The shift from group work to one-to-one encounter, in a more intimate setting and at a non-regularized time, was appropriate in order to meet the patient’s needs. This necessitated a shift in the student’s relationship to therapeutic space, timing and intervention. The Forum discussions broadened to consider situations in which an arts therapist may encounter a patient with whom they are working therapeutically outside of the therapy room or art studio, perhaps in a communal space within the hospice or hospital, for example. Forum members reflected on the need for a human and relational approach during such encounters.

Forum members also reflected that boundaries are meant to serve the work and not the reverse. As students navigate their early palliative care encounters, they may default to conventional notions of boundary setting according to theory taught in their educational programs. Conversely, some may find that the heightened emotional nature of PEoLC adversely affects their ability to set boundaries (Mondanaro & Tsiris, 2021). Practice educators and supervisors play a crucial role in supporting students to understand the role of boundaries while considering the specificities of palliative care and of each individual case.

When an arts therapist notices that their use of boundaries is manifesting as a defense, in a way that is unhelpful to the therapeutic process, this can and should be explored in the therapist’s own self-process in supervision and/or in personal therapy. Such ongoing reflective practice is equally relevant to students and qualified arts therapists. Investment in such work can enable a therapist to better understand and acknowledge the source of feelings and motives that may emerge in the form of defensive boundary setting and countertransference. Deep-seated emotions associated with the therapist’s own life experience, including their cultural background, may prompt discomfort and suffering and this can be particularly true when it comes to working with loss, death and dying. If such emotions remain unconscious and unexplored, as E. M. Meyerson et al. (2020) argue, transferential dynamics can have negative implications not only for patient care but also for the therapist’s own sense of resilience and wellness.

In this vein, colleagues from Mount Sinai Hospital (the Brookdale Department) offered the following:

Reflection

Walls or intense impulses to assert boundaries can trigger self-protective mechanisms that should be explored lest the therapist’s protection of themselves undermine therapeutic connection and compassion for the patient. Walls or impasses that come up in a session contribute to the session dynamics. Within the unconscious dynamics of the relationship between therapist and patient, difficult and painful material can be at play. Somebody’s going to feel it, and so it’s incumbent upon the therapist to engage in personal work to better understand what’s coming up in these moments, and how to explore and engage with it authentically. Such self-awareness can allow boundaries to be navigated in an informed and critical way.

From exploration of boundaries and transferential dynamics, our Forum discussions expanded to consider issues of neutrality, disclosure, authenticity and fallibility in palliative care arts therapies work.

The formation of the therapeutic relationship in inpatient care environments does not always follow a conventional pathway from referral to establishment of a therapeutic contract and initial assessment, such as may be more common in mental health and other settings. Patients do not necessarily make an appointment with an arts therapist. The arts therapist may enter the patient’s space to introduce themselves and the in-house arts therapies provision.

Reflection

The patient, experiencing fluctuations in health, in a state of undress, and physically and mentally less able, is perhaps in the most vulnerable position of their lives. With these elements in mind, patients are offered control through choice-making within sessions at every opportunity. When so much is very obviously out of their control it is imperative to provide space for patients to maintain as much autonomy as possible: firstly, the therapist should enquire if they are willing to have the therapist visit at their bedside, asking basic questions such as, “May I sit?”; “Do you need anything just now?” The therapist must rapidly attune to the patient, assessing verbal and non-verbal cues. Although we are in a healthcare environment, we must remember that this is also the patient’s private space. Cognisance of this helps us to attend to power imbalances, although it may not redress them.

Unpacking these observations in the Forum brought to the surface some distinct features within our respective settings. At St Columba’s Hospice Care, for example, the provision of open group sessions as well as other opportunities for creative engagement (e.g., cultural events, exhibitions and live performances) offer additional opportunities for creative encounters that often interplay with the provision of arts therapies through individual and group sessions. This continuum of creative engagement, ranging from specialist to everyday engagement with the arts (Tsiris et al., 2022b), can offer a supportive framework for the patient as their health condition and needs change over the course of their illness. The therapist’s awareness and clarity around the different ways of working and their varied scope of practice within this continuum is essential.

At Mount Sinai Hospital, the overarching hospital-based culture colors arts therapies provision. Forum members based at Mount Sinai felt that the “medical machine” is the driving force in their hospital environment, resulting in a particular type of medicalized mental health framework that can at times feel at odds with their approach to arts therapies practice. Notwithstanding these factors, some Forum members argued that, on an expressive arts level, they have some capacity to recreate and redesign this framework case by case. This is in line with the reflection below by Forum members from the Mount Sinai Hospital:

Reflection

A large factor here is the palliative care environment, which is non-traditional in terms of mental health treatment, and the lack of control in this environment. Another contributing factor is that of time and timing. Patients don’t have control over their time or their schedule. In this reality, we give patients the opportunity to say “no” or “not this time”. The patient has no control over what’s happening within their body. And they may also experience a lack of control over what happens to them in the hospital setting. The parallel process of what’s happening within a patient’s body in terms of *out of controlness* and the *out of controlness* of the hospital environment has tremendous power in and of itself. Such factors challenge the notions that have defined mental healthcare in the USA. This reality begs the question of ‘who has control over the environment in a hospital-based setting?’ In a way, the tables are turned in that patients aren’t calling and making an appointment with a therapist onsite. The therapist is entering the patient’s space; their world, and so the balance of dynamics such as power and privacy is challenged. The idea of power dynamics is out of the hands of the therapist, and it’s out of the hands of the patient as well, because the power rests in the medical milieu where the *medical machine* is the driving force that *is* the power.

The perceived power of the “medical machine” was particularly relevant to the Forum members practicing within a hospital environment in the USA, and it seemed to differ from people’s experiences of working within the UK hospice care environment, where a more holistic and integrated approach to care was prevalent. These differences within the Forum are perhaps a reflection of wider differences not only between hospital-based palliative care and hospice care, but also between our respective sociocultural contexts. In both settings, however, Forum members argued that arts therapists can support other professionals in their palliative care, using the arts as a reflective tool so that their own experiences of loss can be expressed and processed, and so that organizational power dynamics can be explored and reframed.

### 3.2. Therapeutic Relationship and Authenticity 

The manner in which a therapeutic alliance or relationship might serve a patient or family in a PEoLC context is subject to a variety of factors, including the level of patient cognition and acuity, cultural norms, religious and spiritual beliefs and values. It is also subject to factors such as a person’s history of trauma, degree of trust or mistrust towards healthcare systems and providers, and attitudes shaped by a person’s experiences of power dynamics within those systems. Patients and their families can experience positive affiliations with a variety of clinical staff, and while these may not be characterized as psychotherapeutic relationships, it is important that they be respected as relationships of potentially great significance. The short-term nature of arts therapies work in palliative care was a common theme in our Forum discussions. Forum members recognized that university-based training of students, both in the USA and in the UK was, to some extent, oriented towards arts therapeutic work that takes place over a number of weeks or months, with relative consistency and regularity of sessions. Forum members noted that if there is an assumption that therapeutic relationships can only be forged with a patient or their family when days, if not weeks or longer can be afforded, then a vast domain of possibilities in the provision of PEoLC may go unrecognized. For example, single session encounters with patients or family members, series of weekly session appointments that are interrupted or curtailed by patient illness, and encounters at patient bedsides or in communal spaces can all offer opportunities for therapeutic work.

Navigation of such situations can be challenging for trainee arts therapists. For example, it is understandable that novice practitioners might be more prone to feeling like an intruder if a first encounter with a patient comes at a sudden point of change in their condition or in the final stages of their life. Forum members from the Kravis Children’s Hospital reflected that in pediatrics, periods sometimes referred to as “death vigils”, for example, can either be a time when families will want privacy as they engage in “practices important to the child and family” (Schuelke et al., 2021, p. 11) or a time when the inclusion of music or rituals involving the creation of memory making items such as hand molds or fingerprint charms might be desirable. Forum members reflected on how the therapist’s sensitive and authentic presence is vital in such situations.

Reflection

Such situations call for a high level of agility, sensitivity and quality of presence from arts therapists, as interventions pertaining to legacy and memory making might be appropriate to prioritize. Further to the patient’s ‘ripeness,’ intimacy may happen quickly, calling for authenticity and humanness from the therapist. It would be impossible to do this work effectively without this degree of thoughtfulness. Each session is discrete, and a therapist might only have one opportunity to provide such care.

When expressive arts experiences align with a patient and family’s needs for comfort, connection or meaning making, the therapist who facilitates these interactions is uniquely positioned to “serve a critical role as [they] bear witness to the griever’s pain” (Lichtenthal et al., 2023, p. 608), and to hold space for its expression.

In one Forum meeting, we focused on how a practitioner’s personal beliefs might shape their approach to working with patients in palliative care settings, where existential and spiritual questions are often central to the therapeutic process. Safe space was created to explore beliefs about death, religion, and the afterlife. The discussion highlighted the importance of self-reflection, emphasizing that therapists must be aware of their personal views and ensure these beliefs do not interfere with their professional practice so that the therapist can support the patient in exploring their own beliefs without feeling influenced or judged. In PEoLC, where patients may seek meaning or reassurance about life, death, and what may come after, the therapist’s capacity to be present authentically while keeping a helpful reflective distance is vital.

Reflection

The more authentic an arts therapist can be, the more permission it gives others to be their authentic selves. By extending one’s self-exploration to include not only their professional identity but also themselves fully as people, one may hold that space for patients too. Knowing one’s self is a never-ending exploration and requires ongoing commitment to inner work. This may allow us to recognize when to ‘get out of the way’ to make space for what needs to unfold for the person before us. Indeed, such self exploration should be essential training for all arts therapies practitioners in and beyond PEoLC.

The Forum discussed the role of supervision in supporting arts therapists to process their emotional responses to sensitive topics, helping them navigate the complex intersection of personal beliefs, authenticity and professional neutrality. Through this reflective process, therapists can maintain a compassionate and reflective (dis)stance, ensuring that their own views do not overshadow the patient’s autonomy and exploration. Continuous endeavor for self-awareness, supported by supervision, allows therapists to offer a safe, non-judgmental space where patients can explore existential concerns. While acknowledging professional regulation differences in each country (with the HCPC defining ongoing supervision as core to the standards of proficiency for arts therapists in the UK; HCPC, 2023), the Forum members reflected on the importance of supervision beyond one’s graduation. This is essential to self-preservation and longevity in the profession, even when it presents as a matter of personal choice.

Turning to cross-cultural considerations, the Forum discussions highlighted the importance of cultural sensitivity in arts therapies work. People’s beliefs and rituals related to death and dying are shaped by their social, cultural and spiritual backgrounds. Arts therapists need to work with humility and foster an openness to learning from the patient’s perspective. Beliefs and rituals can play a significant role in patients’ experience of symptom management and dying. They can also be a source of existential anxiety and tension exacerbated by the dominant care approach within a setting.

Vignette by music therapist

A middle-aged man with advanced cancer was refusing medication to manage his pain. Although the use of such medication was common in the ward, such a thing would be against his own spiritual beliefs and commitments. African drum playing was his way of regulating his pain experience. The music therapist would visit and play the drums with him, fostering an environment where he could express himself and manage his symptoms.

Examples such as this one, drawing from past experiences of the Forum members, helped us to consider how the arts therapies can offer a powerful non-verbal space for spiritual exploration. Arts therapies can empower patients to express complex, often ineffable feelings about self, lived experience and legacy that can, at times, sit in juxtaposition to the expected course of treatment.

### 3.3. Curiosity

Alongside questions around neutrality and authenticity, the Forum members explored disclosure and the role of curiosity. Person-centered values, as a key feature of palliative care practice (Fang & Tanaka, 2022), may challenge power imbalances between expert/professional and patient, as well as conventional notions of ‘neutrality’ and ethics in practice and research (Haraldsdottir et al., 2019). Such issues were not straightforward for students, requiring translation of psychological and other theories (as taught in their training programs) to practical application within a PEoLC context. Practice educators supported the students to balance fostering a compassionate and authentic environment with the maintenance of a helpful therapeutic distance.

Reflection

“Why do I want to know what I want to know?” How does disclosure as a two-way process fare in this dialogue? Questions such as “are you married?” or “where are you from?” can come from patients as a colloquial inquiry or as a means of connection. An authentic response is born of therapeutic integrity. Regardless of whether such questions are posed in an arts therapies room or at a bedside, an honest response could be: “I am. What makes you ask?” or “Oh, I’m happy to answer the question, what makes you ask?” Such questions are asked for a reason, and that reason can be explored.

Forum members reflected on their practice as relational work requiring the therapist to be able to acknowledge the person in their totality as a human being. The only way that therapists can do that is for them to be able to give permission to their own sense of humility and vulnerability, including an openness to their own fallibility. Such a stance may not be aligned with the dominant narrative of medically oriented settings, as became obvious in the conversational vignette below by Forum members from the Mount Sinai Hospital.

Reflection

Fallibility, when a therapist is making a therapeutic leap and it doesn’t land, means being able to let it go in favor of another direction. To say to a patient “I’m going to offer something and if it doesn’t make sense to you, let’s leave it, but I want to put it out there”, is to reframe authority in a way that makes it benign in the therapeutic relationship. It is impossible for a therapist to be all knowing. Where is the humanity in that? Holding a therapeutic balance amidst a power-based culture, means being able to bow our heads in humility to say, “what we’re creating here is a relationship, and I can’t know what’s going to work for you or not. I can have an informed guess”. Reframing the whole idea of “treatment” to include humility as in “I may have an idea”, “I may suggest an intervention”, or “I may have something that might be helpful, but I don’t know”, is a way of surrendering to the unknown—which can have multiple layers of meaning when one faces mortality and loss. Relinquishing the systemic power that is bequeathed to a therapist by the ‘medical machine’ becomes a vital component promoting agency and self-determination within the therapeutic relationship.

There are many ways and avenues to ultimately foster a positive therapeutic outcome. Retaining the working therapeutic relationship as a bedrock of arts therapies practice, sound decisions on how we respond and engage verbally and non-verbally rely on our responsibility for ongoing reflective processing of the work and the relationship itself. This responsibility, as the Forum discussed, underpins our understanding, assessment, and potential interpretation of therapeutic materials and dynamics.

Reflection

We’re trained to understand with empathy and to sense if some additional information or request is being conveyed, either consciously or unconsciously, through the patient’s explicit enquiry. Is a question being asked for personal gain? Is it a tactic in conscious or unconscious response to the overarching imbalance of power between therapist and patient? Does it carry with it transferential feelings about people in the patient’s life?

Therapists’ interpretation assessment processes should not overlook theirs or others’ humanity. One’s humanity, however, should be in a constant process of checks and balances with one’s professional skillset and insight. Students may take a risk in assuming that disclosure is by default more ‘human’ and perhaps more compassionate, but there are different ways of being compassionate and they do not all depend on disclosure. Nevertheless, when used skillfully and with clear therapeutic intention, disclosure can deepen or broaden the work.

Whether at a conscious level or not, therapists disclose information all the time. It may not be the details of one’s life, but rather the felt experience of it that comes through as a personal story or energetic presence. Therapists are constantly tapping into these parts of themselves during interactions and in the process they are disclosing personal information, whether consciously or unconsciously. People disclose the spoken and the unspoken during human interaction, and therapists are nonexempt from this human experience. Although the facts or the details may be spared, the nuances of lived experience and intimate feelings that inform a therapist’s empathic presence help to establish the safe space in which the patient’s experience can be shared and understood. Forum members explored how such empathic presence allows a therapist to remain emotionally connected to what is happening in the therapeutic space. As a Forum member put it, “there are elements in being a therapist that ensure that the work is being done in a way that is connected, real, and authentic. Therapists tapping into felt experiences for themselves is what makes this work relational”. 

Maintaining a therapeutic perspective while also being authentic and present was expressed as a challenge that student Forum members commonly encountered. This nuanced navigation of boundaries can feel particularly challenging in PEoLC settings.

Vignette by arts therapies student

An arts therapies student was assisting their practice educator with a tour of the facility with a new community patient. During the tour, the patient had shared about their interests and a little about their life before illness. As part of what would be usual rapport building conversation in other relationships, *I’ll tell you about me, now you tell me a little about you*, the patient asked the student, “What was your first degree?” An innocuous question created an unexpected moment of tension. The student, unsure how to respond, gave a vague answer, deflecting the question. However, when asked, the practice educator, a more experienced therapist, gave a straightforward reply, “I’m a music therapist”.

In this case, the contrast between the student’s hesitation and the practice educator’s confidence highlights the nuanced approach needed in these situations. The question posed was not just about the therapist’s academic background but about establishing a connection and building rapport. However, for the student, answering this question felt like a step into personal territory, raising questions of how much self-disclosure is appropriate in building trust. Deflecting the question left the student feeling inauthentic and as if they had unintentionally rejected the client connection. If the question had been asked during a therapy session, the student felt they would have perhaps been more able to manage the enquiry. The practice educator’s response did not directly answer the question but gave important and relevant information about their role, which was therapeutically necessary for the patient to know. Authenticity does not necessarily (or only) increase in parallel with the degree of disclosure. It is notable that although the student perceived the patient’s question as ‘innocuous’, it could in fact carry several different kinds of significance or meaning (e.g., in relation to the patient wanting reassurance that they were in safe hands). The Forum discussed how these potential meanings could perhaps be explored with the patient in the therapeutic relationship. The vignette above points to a balance between therapeutic perspective and empathic engagement—an issue that was common in the Forum discussions, often in relation to considerations about the therapeutic relationship outside the ‘therapy room’ in PEoLC.

### 3.4. Safety in the Therapeutic Space

Our Forum discussion also focused on safety, drawing on our experiences of arts therapies practice in our respective inpatient care units where the physical space itself poses various challenges including spatial limitations, the sterility and potential austerity of the environment and its aesthetics, and the person’s sense of belonging. The values and beliefs of an individual can impact their desire or ability to engage in a therapeutic relationship, especially in acute illness where the individual’s coping strategies and locus of control may draw on pre-existing family or spousal relationships. In such cases, the primary goals of that individual may be to hold these relationships as central, overshadowing perhaps an openness to engaging with a therapist. For some people, this sense may be amplified further by potential unfamiliarity and unknowns that the arts may represent for them.

Cultural influences and social factors can further define how the offering of therapy in any form will be perceived. A person-centered approach that fosters cultural humility necessitates flexible and responsive ways of working. This requires an openness to understand each person’s worldview and a readiness to practice in a manner that is adaptable in relation to the individual.

Reflection

Meeting with someone in a room that they are most likely experiencing as their own private space while in the hospital—their bedroom; an intimate space—raises a need for consideration. The person may be in their pajamas. They may be laying in bed. They may not even be wearing underwear. When we go into such non-traditional therapy spaces, we need to constantly be looking at what is ethical and defining best practice while remaining open and malleable to connection.

A therapeutic relationship can sometimes foster restoration of a sense of power and agency. Such an approach can be particularly important when a person is living with an incurable illness and may be at the end of life. Forum members reflected that, for the arts therapist, restoring a sense of power in a medical environment may seem like a monumental undertaking because of the structures and schedules by which care is delivered. Facilitating restoration of a sense of agency in an environment where uncertainty prevails falls within our purview as arts therapists. In the PEoLC context, an additional consideration is that affirming agency in a patient who may, to a distressing degree, lack control over what is happening in their body is of great therapeutic potency.

Power and authority are inherent in the organizational structures and procedures of a clinical care setting: restoring a sense of agency within this context can become the base of a strong therapeutic alliance between a therapist and patient. The Forum reflected on how these considerations and a person-centered approach to patient care remain vital not only in the inpatient care setting but also in outpatient and community palliative care (Klinck et al., 2021).

The complex negotiation of such dynamics in the moment-to-moment flow of a session can also challenge the therapist’s attendance to the arc of a therapeutic process. Forum members reflected on how an arts therapies session may be subject to potential interruptions due to the nature of an inpatient environment. The sense of uncertainty and disruption which this may bring can impede the feeling of safety that is essential to processing feelings of vulnerability. Combined with any preconceived stigma about therapy, an environment fraught with unpredictability can prevent the facilitation of a therapeutic space perceived as safe.

Reflection

Creating and rendering a space in which the patient feels listened to and taken care of by a professional is an understood imperative because we don’t want to jeopardize the ability of a patient to be able to say what is most deeply on their mind. “I’m afraid of dying”, expressed within the safety of a therapeutic relationship, may be something a patient would not say to their loved ones, so we want to make sure that we are mediating our own responses and openness within the session flow so that the therapeutic space flourishes.

In this context, the Forum unpacked the idea of intimacy in arts therapies work. The more authentic we can allow ourselves to be with ourselves, the more we can hold that space for others, and the more open somebody might feel to be able to accept that space and share with us.

Reflection

As arts therapists, the more we can do the work of going within our own ‘shadows’ and ‘dark’ places—stepping into our own pain and processing it, acknowledging it and working through it—the more we are able to do that for ourselves, the more easily our presence invites the person we are sitting alongside to do the same. Whether stated consciously or unconsciously, the empathic awareness of pain and what it feels like is often shared by others and can imbue a therapeutic exchange with a sense of mutual human experience. For a therapist’s presence to be able to demonstrate, *I can’t know what your pain feels like to you, but I can be with you in your pain while I am aware of my own experience of pain*, can give people permission to trust and be held therapeutically. This humanized approach to therapy is fluid and applies across myriad scenarios. Conveying to a person that *I can sit with you in your fear*, or *I can sit with you in your despair*, is at the heart of therapeutic witness. The only way that therapists can offer this quality is if they have been able to go to those places for themselves. If therapists can do this holding and processing for themselves, then they may be more able to hold the space for a patient, a family member or a bereaved carer.

These considerations lend weight to the argument that trainee therapists should be engaged in personal psychotherapy as an essential component of their professional development, and that qualified therapists may need to re-engage with personal therapy at different times during their careers.

## 4. Discussion

The nature of PEoLC presents arts therapists with certain considerations and challenges that may be unique compared to other settings of work. The short-term nature of various facets of the work, legacy work, working with the individual and their family, health-promoting palliative care, people’s fluctuating health conditions, and supporting families throughout their pre- and post-death bereavement are all some key components that characterize the uniqueness of this area of practice. Overarching factors, such as whether therapists are providing care in an actual hospice program such as St Columba’s Hospice Care or across consult teams and in embedded programs functioning within a medical center, also influence how PEoLC is understood and delivered. These aspects—with practical implications for one’s practice—are often not emphasized in generic arts therapies training, with the consequence that students and practitioners may not face such questions until they find themselves practicing in a palliative care setting. The Knowledge Exchange Forum presented here invites people to share the inherent nuances of this work amongst staff and students alike, while foregrounding sociocultural differences and nuances in arts therapies practice.

Some of the challenges raised in this Forum have been specific to the inpatient milieu, partly due to the very physical circumstances of this care environment. Perhaps the idea of restoring humanity in the inpatient unit is of relevance as an imminent outcome of such forums dedicated to exploring arts therapies practices in different PEoLC settings, their nuances, and potential obstacles to care delivery. For the patient to feel cared for, listened to and held, and even loved in a Platonic sense (Atkinson, 2012; Natterson, 2003; Rockwell, 2019), the therapist must be able to lean into empathy and compassion. Such a relational stance invites curiosity and willingness to engage with interpersonal dynamics that may be otherwise neglected by the overarching systems in which care is offered. These considerations of arts therapies practice have been vital for our Forum discussions, as have the nuances of how different PEoLC contexts are navigated in order to render a humanized approach to care.

Examining our lived experiences as arts therapies practitioners and students in order to improve and advance our practice is a training imperative. To this end, we have presented emerging insights from our Cross-Cultural Knowledge Exchange Forum, in which a number of features of psychotherapeutic practice have been contemplated with the intention of better understanding the needs of patients accessing PEoLC. Recognizing that many of the people we meet in PEoLC are psychologically typical individuals whose lives have been affected by illness, injury, death or loss necessitates the need for a relational approach and genuine connection rendered with flexibility that may not always be possible, appropriate or helpful in other settings. Our discussions in the Forum explored the idea that reflective and flexible adaptation of some ‘conventional’ features of psychotherapeutic practice is helpful in defining a therapeutic space that is individualized and engendered with humanity. The immanent power dynamics of medical and other organizational environments were addressed in our cross-cultural discussions, including the challenges and possibilities of working in non-traditional psychotherapy spaces. The importance of authenticity, curiosity, and empathy in the therapeutic relationship, as well as the need for the therapists’ own inner work, which includes embracing their own fallibility, emerged as some key components of arts therapies practices in PEoLC. 

Drawing on our Forum experience, we argue that such knowledge exchange initiatives can be catalysts for the development of arts therapies practice in PEoLC. Such dialogue has mobilized the hospice movement since its inception, and in recent years, it has been organized and, at times, formalized further by taking the form of Communities of Practice (Wenger, 2010), Project ECHO networks (an approach that forms a key part of Hospice UK’s strategy; Hospice UK, 2024) and knowledge exchange and transfer initiatives, for example. The latter have been particularly common in the context of research and innovation, often with a focus on addressing gaps between research knowledge and its clinical use (Bückmann et al., 2023; Finucane et al., 2022; Kernohan et al., 2018). Our own Forum experience has also demonstrated the value of such initiatives as a complementary element of generic arts therapies education internationally. Fostering peer reflection, as well as cross-cultural and cross-setting considerations, such forums can promote best practice and sector specific networking and professional development—all of which are fundamental to practice and service development, as well as to research and innovation. 

## Data Availability

The original contributions presented in this study are included in the article. Further inquiries can be directed to the corresponding author.
